# Where are the data linking infant outcomes, breastfeeding and medicine exposure? A systematic scoping review

**DOI:** 10.1371/journal.pone.0284128

**Published:** 2023-04-26

**Authors:** Sue Jordan, Sophia Komninou, Sandra Lopez Leon

**Affiliations:** 1 Faculty of Health and Life Science, Swansea University, Swansea, Wales, United Kingdom; 2 Quantitative Safety & Epidemiology, Novartis Pharmaceuticals, East Hanover, NJ, United States of America; 3 Rutgers Center for Pharmacoepidemiology and Treatment Science, Rutgers University, New Brunswick, NJ, United States of America; American University of Beirut Medical Center, LEBANON

## Abstract

**Introduction:**

Information on the impact of medicines on breastfeeding and the breastfed infant remains scarce. The aims of this review were to identify databases and cohorts holding this information, and pinpoint current information and research deficits.

**Method:**

We searched 12 electronic databases, including PubMed/ Medline and Scopus, using a combination of controlled vocabulary (MeSH terms) and free text terms. We included studies reporting data from databases with information on breastfeeding, medicines exposure, and infant outcomes. We excluded studies not reporting all three parameters. Two reviewers independently selected papers and extracted data using a standardised spreadsheet. Risk of bias was assessed. Recruited cohorts with relevant information were tabulated separately. Discrepancies were resolved by discussion.

**Results:**

From 752 unique records, 69 studies were identified for full review. Eleven papers reported analyses from ten established databases with information on maternal prescription or non-prescription drugs, breastfeeding and infant outcomes. Twenty-four cohort studies were also identified. No studies reported educational or long-term developmental outcomes. The data are too sparse to warrant any firm conclusions, beyond the need for more data. The overall picture hints at 1) unquantifiable, but probably rare, serious harms to infants exposed to medicines via breastmilk, 2) unknown long-term harms, and 3) a more insidious but more pervasive harm in terms of reduced breastfeeding rates following medicines exposure in late pregnancy and peri-partum.

**Implications:**

Analyses of databases reporting on the full population are needed to quantify any adverse effects of medicines and identify dyads at risk of harm from prescribed medicines while breastfeeding. This information is essential to ensure 1) infants are monitored appropriately for any adverse drug reactions 2) inform breastfeeding patients using long-term medicines as to whether the benefits of breastfeeding outweigh exposure to medicines via breastmilk and 3) target additional support to breastfeeding patients whose medicines may affect breastfeeding. **The protocol is registered** with the Registry of Systematic Reviews, no.994.

## Introduction

Establishing health service databases and databanks has been costly in time, energy, and money. Their contribution to pharmacovigilance is considerable, particularly where randomised controlled trials are impossible for ethical and logistical reasons, for example during pregnancy and lactation, and where outcomes are so rare that impossibly large numbers of people would need to be recruited to demonstrate statistically significant differences for such outcomes (for example, many congenital anomalies). However, the value of health service databases holding electronic records of routine care and observational research is limited by the data collected, both the variables recorded and their completeness. Population databases provide insights into the determinants of health and the impact of medicines in pregnancy on infant outcomes, but only five European databases have any data on breastfeeding: the national databanks for Finland, Scotland and Wales, EFEMERIS / POMME in Haute-Garonne, and hospital records of breastfeeding at discharge in Tuscany [1].

Breastfeeding is complex, with nutritional, immunological, and psychosocial aspects, which are not easily disentangled. It profoundly affects women and children. Benefits to infants include reduced: mortality (particularly necrotising enterocolitis and sudden infant death syndrome), gastro-intestinal and respiratory infections, acute otitis media, asthma/wheezing [2, 3], malocclusion, obesity and type 2 diabetes. Benefits to mothers include reduced rates of breast and ovarian cancers, type 2 diabetes, myocardial infarction and hypertension [2]. In the USA 3,340 (95% confidence interval 1,886 to 4,785) annual excess deaths are attributed to shortened duration of breastfeeding (defined as less than 1 year, and exclusively <6 months): 78% of these excess deaths are maternal, and 22% infant [2].

The safety of a medicinal product during lactation is complex, in that it involves the effects of medicines on both infant and mother plus the interactions and bonding between them. Their very different pharmacokinetics (particularly elimination half-lives), and the need to calculate these for mother, neonate, and preterm neonate complicate determination of safety. Before a medicine’s safety profile can be considered complete, several questions need to be addressed:

- How does the medicine affect the physiology of lactation?
- How are breastfeeding rates affected by administration during pregnancy, labour, the puerperium and during lactation?
- Can these effects be mitigated by recognition, support, and clinical management?

What is the effect of the medicine on the breastfed infant? Some 70% infant ADRs are dose-dependent [4], but concerns remain regarding preterm infants and those with allelic variations in key enzymes–the extreme phenotypes [1].

- How should the infant be monitored for any possible adverse effects?
- Do the benefits of breastfeeding outweigh possible disbenefits from exposure to medicines via breastmilk, short- and long-term?

Currently, studies reporting breastfeeding, its predictors and consequences are, with few exceptions, based on recruited cohorts [5]. Some existing cohorts with potential for pharmacovigilance, such as the Millennium Cohort Study [6], the Norwegian and Danish mother and baby studies [7, 8], are linked with population databases. Without full population coverage it will be difficult to report associations free of volunteer [9], and collider bias. These arise when samples do not represent the population, because volunteering is related to variables being investigated, such as medicines exposure, breastfeeding or social class [10–14].

Although population databases are an important advance in pharmacovigilance, it appears that they may be less than comprehensive, particularly for issues affecting women and children, including pregnancy prevention programmes aiming to reduce exposure to known teratogenic medicines [15]. If pregnant and breastfeeding women and children are not to be excluded from global pharmacovigilance initiatives, population databases with information on breastfeeding for the full population should be identified. We defined a database as “a structured set of data held in computer storage” [16], more specifically, a large collection of data organized and maintained so that it can be expanded, updated, and retrieved rapidly for various uses [17]. To inform the discussion around implications for practice, information from cohort studies [18], not derived from databases, was tabulated. This systematic scoping review aimed to identify and report the databases and cohorts with information on breastfeeding and its impact on infants, and summarise any apparent information and research deficits.

## Method

We conducted a scoping review using systematic searches to map and locate the databases providing quantitative evidence on medicines exposure, breastfeeding and infant outcomes, and summarise the evidence [19, 20].

The protocol for this search is registered [21] (S1 File). This review follows the PRISMA guidelines [22], and the extension for scoping reviews [23].

### Search strategy

Twelve electronic databases (PubMed/Medline, Scopus, CINAHL, PsycINFO, Web of Science, British Nursing Database, Proquest, Drugs and Lactation Database (LactMed), ZETOC, TRIP, MIDIRS, Wiley Online Library) were searched to May 2022 using a combination of controlled vocabulary (MeSH) and free text terms. These included terms for breastfeeding, lactation, or infant feeding along with terms for pharmacovigilance or drug monitoring or drug surveillance. The search strategy is shown below. There were no language or date or location restrictions, but the search was restricted to papers reporting on humans only.

#### Search strategy

*Search terms*. “Breastfeeding OR Lactation OR Breastfe* OR Breast-fe* OR “Breast fe*” OR Lactat* OR “Infant feed*” OR “Infant Nutrition”

AND

“Pharmacovigilance OR Product Surveillance OR Postmarketing OR Drug Monitoring OR Adverse Drug Reactions OR Pharmacovigilan* OR “Drug monitor*” OR “Postmarketing Surveillance” OR “Post-marketing Surveillance” OR “Post marketing Surveillance” OR “Adverse Drug Reaction*”

NOT

For two databases (PubMed and PsychINFO) it was necessary to specify “NOT economics” in order to obtain more relevant results.

### Inclusion and exclusion criteria

#### Inclusion criteria

Reports from databases or cohorts with empirical data on breastfeeding plus human maternal medication exposure plus infant outcomes plus pharmacovigilance or adverse drug reactions.

#### Exclusion criteria

- No empirical data on infant feeding/breastfeeding, maternal medicine exposure and infant outcomes / welfare.
- Single case reports.
- Cross sectional surveys
- Articles not in English with neither an English abstract nor empirical data tables.

We excluded pharmacokinetic plasma / milk transfer studies and case series where there was no information on infant outcomes. Where infant outcomes were reported, we included these cohorts. Literature reviews were excluded but reference lists were examined for further databases. We excluded papers a) without empirical data and b) not reporting infant outcomes. Tabulation aimed to describe the database or cohort (size, location) and participants, exposures or interventions (medicines, doses and timing), outcomes (breastfeeding rates and infant welfare or ADRs), and the inferences of the investigators. After initial data extraction, some items were collapsed where there was a paucity of information, for example on comparators and long-term outcomes.

### Study selection

Following the search, duplicates were removed, and publications were screened by titles to identify those likely to meet the study inclusion criteria. This was carried out independently by two blinded researchers (SJ/SK or SLL/SK). The titles and abstracts or first pages of the remaining studies were reviewed by two researchers, blinded (SJ/SK or SK/SL) according to inclusion and exclusion criteria. Papers were then selected for full review. Full texts of all articles selected for consideration were retrieved, read, and decisions on inclusion were reached jointly. The reference lists of included studies were reviewed to identify other possibly relevant studies. These studies were then reviewed following the same process outlined above. We separated the studies reporting established databases from those reporting recruited cohorts. The relevant details from included papers were tabulated and checked independently (by SJ and SK and re-checked by SLL) (Tables 1 and S1).

**Table 1:** Studies using databases reporting medicines use during breastfeeding (chronological order).

Reference Database/ Location/ Dates	Number of participants	Infant ages	Medicines	Doses	Impact of medicine on breastfeeding	Impact on infant if Breastfeeding	Other Outcomes	Trimester medicine used
**Multiple medicines**								
(Kronenfeld et al., 2018) / Drug Consultation Centre for pregnant or lactating women (DCC)/ Israel/ Jan2011—Dec 2015. [35]	547 of 626 BF women consented, 395 (72.2%) sought information on psychotropics, and 152 (27.8%) on antibiotics. 115 women prescribed psychotropics were excluded (polytherapy 35, unmedicated 27, not BF 53).	Median age of the infants in the psychotropic group at follow up was 20 (11–33) months and 36 (20–48) months in the antibiotic group.	193 SSRIs, 37 benzodiazepines, 23 SSRIs, 11 TCAs, 6 1^st^ generation antipsychotics, 5 2^nd^ generation antipsychotics, 2 other antipsychotics, 2 norepinephrine reuptake inhibitors. 58 metronidazole, 48 new macrolides, 22 quinolones, 12 nitrofurantoin, 7 fosfomycin, 4 doxycycline, 1 clindamycin, 1 cefuroxime.	Reported as within recommended ranges.	Psychotropics were associated with shorter BF duration (median 24 weeks) and lower exclusivity rates (35%) compared with antibiotics (median 36 weeks and 61% exclusively). 13 (4.6%) cases prescribed psychotropics and 5 (3.3%) prescribed antibiotics reported low breast milk production.	• 14 (5%) infants exposed to psychotropics and 7 infants (4.6%) to antibiotics experienced ADRs. • Antibiotic exposure was associated with diarrhoea (7 vs. 0 infants). • Psychotropics (SSRIs, lorazepam, and amitriptyline) exposure was associated with sleepiness in 8 infants and was observed shortly after birth in 6 of the cases, (reported by mother) (escitalopram- 2 cases, paroxetine, amitriptyline, lorazepam, and sertraline, 1 case each) and further one infant (escitalopram) at 3 days, and another (citalopram) at 4 days. All sleepiness resolved spontaneously within 24 hours.Gross developmental milestones reported as normal in all infants.	No differences between groups in pregnancy complications. More neonatal complications and foetal distress was reported in the psychotropic exposed: 19 vs 1 and 15 vs 0 respectively.	Not fully specified. All trimesters for the infants reported as ‘sleepy’.
(Soussan et al., 2014) / French national Pharmacovigilance Database/ France/ 1984–2011 [30]	174 cases reported. Voluntary spontaneous reporting rate unknown.	Mean age 7.0 (SD 9.5) weeks [range 1 day–2 years]; 63% <1 month, 37% 1 month to 2 years.	In order of frequency of reporting: Paracetamol (usually in combination), Dextropropoxyphene, Hydroxyzine, Ketoprofen, Amoxicillin + clavulanic acid, Ascorbic acid, Lamotrigine, Valproic acid, Levonorgestrel, Ibuprofen, Flavinoids, Iron, Clonazepam, Amoxicillin, Pseudoephedrine, Carbamazepine, topiramate, Clorazepate	Not reported	not reported	65 (37.4%) ADRs were serious. Most frequently reported and serious ADRs concerned behavioural problems, sedation, insomnia), diarrhoea, and vomiting). • Dextropropoxyphene was implicated in: hypotonia, apnoea, respiratory distress, bradycardia, weight loss and constipation. • Hydroxyzine in sedation. • Ketoprofen in oesophageal ulcer, erosive gastritis, meningeal haemorrhage and renal insufficiency. • Lamotrigine in sedation, hypotonia and weight loss. • Benzodiazepines were implicated in hypotonia, apnoea and somnolence. • Single cases of neutropoenia associated with carbimazole, vomiting with interferon-alpha, bradycardia with propranolol. ADRs resolved in 79.3% cases. Outcome unknown in 20.7%.	Not reported	Exposure during breastfeeding reported. Use in pregnancy not reported.
(Ito et al., 1993)/ Motherisk programme/ Canada/ Jan 1988 to June 1991 [38]	1110 from a TIS database. 272 out of 1110 excluded due to no drug exposure, leaving 838	up to 3 months	116 antibiotics 196 analgesics 85 antihistamines 42 sedatives, 6 carbamazepine 16 oral contraceptives 5 warfarin 166 used multiple medicines	Not reported	3 of 16 mothers taking oral contraceptives thought that their milk volume was slightly decreased. 36 temporarily ceased BF, and 18 permanently ceased BF, due to concerns.	94 mothers (11.2%) reported infants’ minor adverse reactions not requiring medical attention: • antibiotics (19.3%) (diarrhoea), analgesics or codeine (11.2%) (drowsiness), • antihistamines, (9.4%) (mainly irritability) • sedatives, antidepressants or antiepileptics (7.1%) • others (9.9%).	No major ADRs necessitating medical attention were observed.	. Use in pregnancy not reported.
**Single drug or medicine**								
Kaplan et al 2022 / worldwide manufacturer’s database in Israel, but most BF data from Germany, Canada and Turkey. / 2019–2021 [33]	2327 pregnancies on database, 1406 known pregnancy outcomes, (1433 foetuses), 393 followed to 1 or 12 months, 18 with infant and BF data.	Up to 12 months	Glatiramer acetate for MS, by subcutaneous injection of 1 ml prefilled injection.	20 mg/ml od, 40 mg/ml thrice weekly and both.	75/ 393 (21.2%) breastfed at 1 month. 169/1182 (14.3%) live births BF whilst taking glatiramer. At 12 months, 40 women reported any breastfeeding. Mean duration 7 (SD 4.3) months.	• No developmental delay was reported in the whole database. • Adverse events and hospitalisations were not analysed by breastfeeding status. • Height and weight gain amongst breastfed infants appeared within normal limits.	1202/1433 (83.9%) foetuses live born. Gestation and birth weight reported for 415 and 399 infants. Adverse events were reported for 67/354 (18.6%) live births.	All trimesters
(Ko et al., 2018) / Pregnancy Risk Assessment Monitoring System (PRAMS)/ Alaska, Hawaii, and Vermont, US/ 2009–2011 [29]	4969 women *post-partum*	Not reported	Marijuana (cannabis)	Not reported	Postpartum marijuana users were more likely to breastfeed for < 8 weeks (34.9% vs. 18.1%).	No reports of impact on breastfed infant.	Postpartum marijuana users were more likely to smoke cigarettes (48.7% vs. 20.3%), and experience postpartum depressive symptoms (14.0% vs. 9.0%). Prevalence of low birth weight and preterm birth were similar.	Not reported.
(Crume et al., 2018) PRAMS / /Colorado, USA / 2014–2015 [39]	3207 women *post-partum*	Not reported	Marijuana (cannabis) The self-reported prevalence of cannabis use at any time during pregnancy was 5.7 ± 0.5% and the prevalence of early postnatal cannabis use among women who breastfed was 5.0% (95% CI, 4.1%-6.2%)	Not reported	Pre- and post-natal cannabis use were associated with shorter BF duration 88.6% prenatal cannabis users (95% CI, 80.8%-93.5%) initiated BF, as did 93.8% non-users (95% CI, 92.5%-94.9%). 64.4% (95% CI, 54.9%-72.9%) prenatal users BF for ≥9 weeks as did 78.3% (95% CI, 76.2%-80.3%) non-users. 57.6% (95% CI, 47.4%-67.2%) postnatal users BF for ≥9 weeks as did 78.7% (95% CI,76.6%-80.6%) non-users	No reports of impact on breastfed infant.	Prenatal use was associated with a 70% increased likelihood of small for gestational age (95% CI, 1.1–2.6); however, the relationship was not statistically significant after adjustment for prenatal tobacco use. The likelihood of NICU admission and preterm birth was not significantly increased for mothers who used cannabis during pregnancy.	All trimesters
(Brunner et al., 2013)/ Lilly Safety Database/ Global/ Sept 1986 –Dec 2010 Data from spontaneous reports, clinical trials, post-marketing observation. [34]	610 pregnancies exposed to olanzapine, 102 exposed while BF. 62 reported doses. 30 reported BF duration.	Not reported	Olanzapine (oral)	Range during BF: 2.5–20.0 mg/day (mean 7.4) Oral dose reported in 535 (87.7%) pregnancies, range: 0.6 to 35.0 mg/day (mean 10.3) injections in <1%.	Not reported BF duration ranged 2 days to 13 months (mean 74 days).	16 (15.6%) reported an adverse event in the infant with temporal association with breastfeeding: most commonly somnolence (3.9%), irritability (2%), tremor (2%), and insomnia (2%).Infants reported as: • recovered/ recovering after 40% of events, • not recovered in 24% of events, • unknown outcome in 36% of events.	401 (66%) normal births, 60 (9.8%) premature births, 57 (9.3%) spontaneous abortions, 49 (8%) perinatal conditions, 27 (4.4%) congenital anomalies, and 16 (2.6%) other outcomes (including ectopic and stillbirth).	All trimesters
(Gilad et al., 2011)/ Beilenson TIS / Israel/ Mothers seeking information 2005–2008 were contacted 1–2 years after initial query.[36]	88 women contacting a TIS. 37/70 exposed to olanzapine (22 breastfed). 51 exposed to paracetamol.	Up to 1–2 years	Olanzapine	Mean daily dose 6.24 (SD 4.10) mgs	15/37 did not initiate BF, 2 due to difficulties, 4 due to fear, 5 on medical advice, 4 unclear. Early BF discontinuation was more common in olanzapine-exposed dyads (5/ 22 vs 0/ 51 taking paracetamol). Little difference in duration of BF.	Of 22 olanzapine-exposed BF infants: 3 experienced ADRs, 2 failed to gain weight, 1 had speech delay, and 1 motor delay (1 infant had 2 problems). 3 /51 infants exposed to paracetamol failed to gain weight, and 1 had feeding problems.	8 of 30 neonates exposed to olanzapine late pregnancy had problems: 3 withdrawals, 2 respiratory distress, 1 hypotonia, 2 poor sucking or feeding difficulty. 1 / 51 exposed to paracetamol had a problem.	All trimesters for 18 of 22 exposed women
(Goldstein et al., 2000)/ Lilly Worldwide Pharmacovigilance Safety Database / all identified maternally exposed cases with outcomes reported to the database from first human dose with olanzapine until October 1, 1998. [31]	50 women in total: 2 breastmilk exposures were identified retrospectively. 37 pregnancies identified prospectively 11 retrospectively.	At birth: 1 infant (exposed in pregnancy), at 2 months: 1 infant (not exposed before birth)	Olanzapine	The 2 BF women received 5 and 10mg/day. Doses were reported for 30 pregnancies. Range: 5- 25 mg/day, mean daily dose was 12.9mg. Median 10 mg/day	2 mothers reported: 1 mother substituted infant formula at 7 days, but symptoms did not improve. The other continued breastfeeding.	There were two retrospectively identified lactation exposures. • One report involved an infant with cardiomegaly. Although bottle-feeding was initiated on the seventh day, jaundice and sedation continued. • The second infant was exposed at 2 months when the mother began treatment for schizophrenia with olanzapine 10 mg/day. The mother was also taking paroxetine, trifluoperazine, and procyclidine. The infant experienced no adverse events.	Of 37 prospectively identified: 14 induced abortions, (1 ectopic pregnancy), 3 spontaneous abortions, 1 stillbirth (pregnancy complicated by gestational diabetes, thrombocytopenia, hepatitis, and polydrug abuse). Of 11 retrospectively identified pregnancies, 3 infants died, 2 had congenital anomalies and 5 had perinatal complications.	All trimesters
**Single group of medicines**								
Noseda et al 2021 Vigibase, Uppsala monitoring centre, Sweden / WHO pharmacovigilance database of spontaneous reports from all reporting centres, mainly USA / inception to end 2019 [32]	94 safety reports for medicines in question, 1 of poor breastfeeding. 21,149,392 total safety reports.	Not reported	Monoclonal antibodies for migraine: erenumab, galcanezumab, fremanezumab Co-exposures reported for some outcomes.	Not reported	No information on BF rates. A single case report of poor feeding.	Not reported	23 cases of spontaneous abortion reported, (5 had co-exposures). Reporting odds ratio compared with triptans 1.85, 1.12–3.13.	All trimesters
Veiby et al 2013 MoBa /Norway/ mid-1999 to Dec 2008 [28]	223 women using AEDs from MoBa cohort, linked with national medicines databases. 276 women with epilepsy and no AED (total 499). 77,770 reference children	6, 18 and 36 months	AEDs Antiepileptic monotherapy (182): carbamazepine (48, lamotrigine (71), valproate (27), other 36. Polytherapy: 41.	Not reported	BF rates varied within groups and were lowest with lamotrigine monotherapy. Compared with referents, exclusive breastfeeding was less common among women using antiepileptics at 6 months (46% vs 56%). More women using AEDs were not BF at birth (13% vs 3.6%) and 6 months (33% vs 19%).	Impairment in fine motor skills occurred in 4.8% (3648/77,770) of referents, 11.5% (25/217) of infants exposed to AEDs, 8.3% (12/148) of infants exposed to AEDs and breastfed. Fine motor and social skills were more likely to be impaired if mothers had used AEDs in pregnancy (OR 2.1, 1.3–3.2). Where epilepsy was unmedicated, the difference was less (OR 1.4, 0.8–2.2).	Continuous breastfeeding during the first 6 months was associated with a tendency toward improved outcomes for all the developmental domains regardless of maternal AEDs. Not BF was associated with increased risks of autistic traits (OR 3.0, 1.2–7.4) for children exposed to AEDs *in utero*.	All trimesters
(Gorman et al., 2012) / California Teratogen Information Service Clinical Research Program (CTIS) / California, US/ January 1st, 2000 to June 1st, 2010 [37]	466: 167 exposed to SSRIs at birth, 117 exposed earlier in pregnancy, and 182 not exposed to SSRIs, enquiring about paracetamol or dental treatment.	2–4 weeks	SSRIs: Citalopram Escitalopram Fluoxetine Fluvoxamine Paroxetine Sertraline	Median daily dose (mgs) Citalopram (20) Escitalopram (10) Fluoxetine (20) Fluvoxamine (25–300) Paroxetine (20–22.5) Sertraline (50–75)	Of women not using SSRIs, 90% initiated BF and 65% were fully BF at 2 weeks. Of women using SSRIs at birth, 79% initiated BF, and 51% were fully BF at 2 weeks postpartum. Among those discontinuing before birth: 81% initiated BF and 52% were fully BF at 2 weeks.	Not reported	SSRI exposure was associated with: Length <10^th^ centile. Other differences (e.g. NICU use) did not reach statistical significance.	All trimesters

Notes to table: the detail reported varied between the papers. AEDs = anti-epileptic drugs, BF = breastfeeding, CI = confidence intervals, OR = odds ratio, PRAMS = Pregnancy Risk Assessment Monitoring System, SD = standard deviation, SSRI = selective serotonin reuptake inhibitors, TCA = tricyclic antidepressant, TIS = teratology information service.

Tabulated information was summarised, in accordance with the review’s objectives to describe the databases reporting on medicines, breastfeeding and infant outcomes simultaneously, and report the purported effects of medicine exposure [20]. A critical appraisal of the risk of bias in the database reports was based on a recognised tool for assessment of non-randomised studies of exposures using consensus-driven domains relating to: confounding, selection, intervention misclassification or mismeasurement, post-exposure interventions, missing data, measurement and selective reporting (ROBINS-E) [24]. We were unable to assess the direction of bias (SJ, checked by SK, SLL).

## Results

Searches identified 858 titles. A further four studies were identified by reviewing the reference lists of included studies, a total of 862. Removing duplicates reduced numbers to 752. We were unable to identify an abstract for 52 of these, so they were reviewed by title, date and provenance. First page or pdf was identified for 35. Seventeen were book chapters, 11 were editorials, 7 were clinical notes. Seventeen, had neither first page nor abstract. Ten studies, dated from 1966 to 1999, had no email contact details. We contacted the remaining seven authors but received no responses.

**Fig 1:**
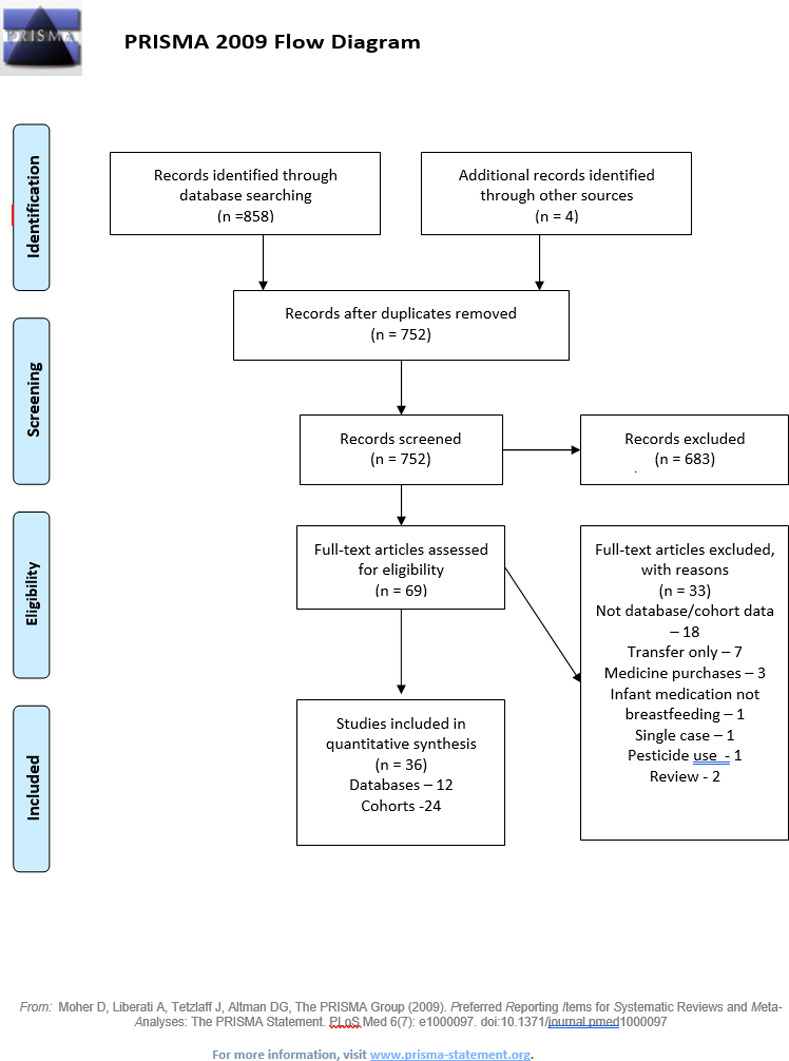
Selection of studies: Flow diagram.

Review of titles and, if needed, abstracts or first pages of the remaining 700 studies identified 69 papers for full review. The most common reasons for exclusion were: ‘out of scope’, absence of empirical data (mainly reviews), and absence of data on infant outcomes or welfare. We excluded 14 papers that could not be retrieved in full and pre-dated 2004, the year of the earliest database identified in our earlier work [1]. 36 papers had neither an English abstract nor empirical data tables. Of the 69 papers initially identified for further review, 33 were excluded as they did not meet the inclusion criteria (for detailed exclusion reasons, see PRISMA diagram, Fig 1), leaving 36 studies (Fig 1). Most (18) excluded papers did not contain data from databases or cohorts, seven described the transfer of medicines into breastmilk but did not report on infant outcomes, even to say that infants were well. Three database studies described purchase of medicines, and one infant medication. The paper on the negative impact of pesticides on breastfeeding rates [25] was considered ‘out of scope’. Of the two papers with information on dose-response one was a single case report [26], and excluded; the other report emanated from a cohort of 7 infants (S1 Table) [27] All papers with more than one participant with relevant data were tabulated. Details of all included database studies were extracted including study objectives, study location, and details of exposures, participants, outcomes, and findings (Table 1).

### Databases identified

Ten databases were identified (Table 1). We included the Norwegian MoBa cohort in this table, as it is linked to the database of Norwegian national records of medicines dispensed in primary care. However, data on breastfeeding were only available for patients linked with a volunteer prospective cohort study [28]. The PRAMS database contains whole-population data on infant outcomes, but marijuana (cannabis) use was taken from self-reported questionnaires from a stratified sample of live births across the USA and links with birth certificate information [29]. Spontaneous reports formed the basis of five studies and four databases [30–34]: two of these studies were from the olanzapine manufacturer’s databases, and one from glatiramer acetate manufacturer’s database [33]. We identified only one national database reporting adverse drug reactions (ADRs) in breastfed infants: the French spontaneous reporting database [30], and only one report of an adverse event affecting breastfeeding in the Uppsala Monitoring Centre international database [32]. Four medicine information centres generated databases based on patients’ spontaneous contacts followed up by telephone to ascertain outcomes: two in Israel [35, 36], and one each in California [37], and Canada [38]. The Centre for Disease Control’s (CDC’s) Pregnancy Risk Assessment Monitoring System (PRAMS) was used to report data on recreational drugs in two papers [29, 39].

Three papers emanated from olanzapine surveillance: two from manufacturers [31, 34], and one from an information service [36]. Two papers from one database reported on recreational drugs [29, 39]. Four reported on specific drug groups: psychotropics (any) [35], SSRI antidepressants [37], antiepileptics [28], monoclonal antibodies for migraine [32]. Only two papers reported use of any or all medicines [30, 38]. Three compared exposures to psychotropics with other medicines: antibiotics [35], paracetamol or dental treatment [37], paracetamol [36]. [28] compared outcomes for those using AEDs with unmedicated epilepsy and the reference populations. [29, 39] compared outcomes for those using or not using marijuana. Nine papers reported on both pregnancy and breastfeeding exposures [29, 31–33, 35–38]. Psychotropic medicines, but not marijuana, appeared to be associated with a relatively high incidence of suboptimal perinatal outcomes, including withdrawal reactions and poor suckling [36].

Six databases reported on the impact of prescription medicines on breastfeeding [28, 29, 31, 35–38]. No databases related to hospital prescribing, although spontaneous reports did not specify the provenance of prescriptions. None held information on medicines in labour.

From ten papers, data on 4,264 dyads exposed to prescription medicines were reported, and two further papers reported on 8,176 dyads surveyed regarding marijuana (cannabis) [29, 39] (total 12,440).

### Cohorts identified

We identified 24 papers reporting recruited cohorts, each describing a single study. These are presented in S1 Table. Cohorts reporting infant outcomes, maternal medicines exposures and breastfeeding ranged in size from three to 1719 infants: seven included less than 10 infants. One large cohort represented follow up from two randomised controlled trials (RCTs) in Botswana: the cumulative incidence of severe anaemia in breastfed infants varied between antiretroviral regimens [40]. Nine cohorts reported on breastfeeding as an outcome, and all 24 on infant outcomes. No studies reported using breastfeeding as a covariate.

### Impact on breastfeeding rates

In cohorts and databases, prescription medicines, marijuana and pesticides adversely affected breastfeeding rates. Reasons for shortened breastfeeding duration included: patients’ concerns over prescription medicines [38, 41], weak suckling [42] or adverse effects on the infant [43, 44]. Decreased lactation following oestrogen and progesterone exposure led to early discontinuation of breastfeeding [38, 45]. Early discontinuation of breastfeeding was also associated with mental health medicines [35], including olanzapine [36], SSRIs [37], antiepileptics [28, 42].

### Impact on infants

The French pharmacovigilance databases provided whole-population data, but relied on spontaneous reports, which may underestimate ADR prevalence by over 90% (Hazell & Shakir 2006). Selection, volunteer, and collider bias were not reported in any papers. Medicine exposure via breastmilk affected some, not all, infants. Two databases and one cohort reported that some infants exposed via breastmilk experienced serious ADRs, mainly the known adverse effects of medicines [30, 36, 40]. For example, following exposure via breastmilk there were cases of: infant apnoea following maternal use of benzodiazepines or opioids: haemorrhage and infant renal insufficiency following ketoprofen; and neutropoenia following carbimazole [30]. There were single case reports of hypotension associated with a beta blocker [46] and impaired suckling and vomiting with carbamazepine [42].

Some, but not all, infants whose mothers took benzodiazepines or opioids [30, 38, 44, 47], olanzapine [34] or other mental health medicines [35, 48], were sedated or sleepy or constipated [48], which may have led to failure to gain weight due to insufficient feeding [36]. Infants exposed to SSRIs were more likely to be irritable and/ or feeding poorly [43, 49], but this was not reported in all studies [37], particularly the small cohorts [49–51]. Dose were not always reported, and there were no dose-response analyses. The only exploration of the effect of dose was a report of olanzapine dose reduction resolving drowsiness for one infant [27]. Similarly, reducing the dose of citalopram improved infant sleep in a separate single case report [26].

‘Minor’, well-known adverse effects were pervasive, affecting 94 out of 838 infants [52]: these included infant diarrhoea following maternal antibiotics [35, 52] or antipsychotics [48], and infant oral *Candidiasis* following metronidazole [53]. Two papers from one database reported no adverse effects in infants exposed to marijuana [29, 39].

There were no reports of educational outcomes or follow up beyond 3 years. Twelve studies reported various developmental outcomes [28, 35, 36, 49, 50], including six small cohorts (with <11 participants) [27, 51, 54–57]. One of six infants [27], three of 22 [36] exposed to olanzapine, five of 28 exposed to olanzapine, risperidone or quetiapine [48], and one of 10 exposed to antidepressants [54] exhibited developmental delay. Following *in utero* exposure to antiepileptics, breastfed infants were less likely to exhibit autistic traits than formula fed infants [28]. Four of 10 infants exposed to lithium had abnormal results for renal or thyroid function in venous blood samples, but no other observable ADRs; long-term sequelae were not ascertained [56].

### Risk of bias

Most analyses were descriptive, and based on biological plausibility. Few analyses accounted for all known confounding variables, such as socio-economic status (SES), alcohol use [58], or pesticide exposure [25], despite known associations. The impact of fluctuations in milk composition and fat content or the potential for increased exposure associated with clinical or subclinical mastitis were not discussed [59, 60]. No studies defined the extent of breastfeeding, whether exclusive or partial: recent studies relied on self-report [33, 48, 61]. All studies relied on maternal self-report of breastfeeding: this may over-estimate duration [62] and initiation of breastfeeding [63], but is considered reasonably accurate if recalled within 3 years [64].

Only one study [40] involved randomisation: this is likely the definitive work on exposure to highly active anti-retroviral therapy (HAART) regimens, but it did not involve a database. It was the only cohort study with >200 participants. The six databases from information services [32, 33, 35–38] were vulnerable to bias emanating from self-selection and non-response to follow up. The PRAMS database was vulnerable to non-response bias, despite exhaustive attempts at telephone contact, and the MoBa recruited cohort to volunteer bias. The databases relying on spontaneous reports [30, 34], were crucial in signal generation, but may fail to identify the majority of ADRs.

Although the database studies were well-conducted, these inherent limitations in their design puts them at moderate risk of bias, at best (Table 2).

**Table 2:** Appraisal of risk of bias in databases identified (chronological order).

Reference Database/ Location/ Dates	N / type of study	Risk of bias due to confounding	Risk of selection bias	Risk of bias in exposure measurement	Risk of bias due to post-exposure interventions	Risk of bias due to missing data	Risk of bias in measurement outcomes	Risk of bias due to selective reporting	Overall assessment
**Multiple medicines**									
(Kronenfeld et al., 2018) / Drug Consultation Centre for pregnant or lactating women (DCC)/ Israel/ Jan2011—Dec 2015. [35]	547 women / BF 395 (72.2%) sought information on psychotropics, and 152 (27.8%) on antibiotics. 115 prescribed psychotropics were excluded. Convenience sample of women seeking information.	Moderate Confounding by indication may have occurred. Women using multiple medicines or unmedicated were excluded. Propensity scores used as covariates.	Unknown Self-selected sample of women contacting an information centre.	Unknown Self-report of medicines use.	Unknown Self-report of medicines use.	Unknown Self-selection and self-report.	Unknown Women were telephoned for self-reports.	Moderate Women self-reported.	Moderate risk An important exploratory study, vulnerable to volunteer bias. When covariates were accounted, the finding on sleepiness remained important.
(Soussan et al., 2014) / French national Pharmacovigilance Database/ France/ 1984–2011 [30]	174 cases reported. Voluntary spontaneous reporting rate unknown.	Unknown No information on confounding by indication and covariates.	Unknown Women may have self-selected when reporting ADRs.	Low Routine data used.	Moderate Medication histories obtained from routine data, but no information on recreational drugs.	Unknown The prevalence of unreported cases is unknown.	Low Physician assessed.	Moderate Only some 5% ADRs are spontaneously reported. Minor ADRs are rarely reported.	Moderate risk Whole population surveillance of adverse events. Important information.
(Ito et al., 1993)/ Motherisk programme/ Canada/ Jan 1988 to June 1991 [36]	838 Convenience sample of women contacting an information service.	High No information on confounding.	High 1110 of 2018 self-selected initial callers were re-contacted by telephone for information.	Unknown Self-report of medicines use.	Unknown Self-report of medicines use.	High Low re-contact rate.	Unknown Women self-reported	Moderate Women self-reported.	Moderate risk An important exploratory study indicating the need to explore possible harms.
**Single drugs or medicines**									
(Kaplan et al., 2022) / worldwide glatiramer’s manufacturer’s database in Israel. / 2019–2021 [33]	2327 pregnancies 1406 known pregnancy outcomes, 393 followed to 1 or 12 months, 18 with infant + BF data.	High. No information on covariates or confounding.	Unknown Self-selected and spontaneous reporting of adverse events by women and professionals	Unknown Information on exposure mainly from self-report	Unknown Information on exposure mainly from self-report	High 18 / 2327 pregnancies had data on breastfeeding at 12 months.	Unknown Women self-reported BF.	Moderate Reliance on spontaneous and self-reports. Information on neurodevelopment not sought.	High risk for BF outcomes. Study and database funded by manufacturer.
(Ko et al., 2018) /PRAMS/ Alaska, Hawaii, and Vermont / 2009–2011 (marijuana [cannabis]) [29]	4969 women post-partum. Stratified population sample received questionnaires plus telephone follow up for non-response, linked to birth registration data.	Low Covariates analysed.	Moderate Response rate to questionnaires not reported, but non-response bias likely.	Moderate Marijuana use was self-reported, but higher than expected, given that recreational use was illegal at time of data collection.	Unknown Neonatal outcomes and maternal health data were obtained from databases, but little information on concomitant prescriptions reported.	Moderate Unknown non-response rate. 10% subjects had missing information.	Unknown Data from self-reported questionnaires and birth certificates for some demographics.	Low Reporting comprehensive.	Moderate risk Volunteer questionnaire respondents linked with population data.
(Crume et al., 2018) PRAMS/ /Colorado / 2014–2015 (marijuana) [39]	3207 *post-partum* women PRAMS study, as for Ko et al, above	As above (same database and methods)	As above	As above	As above	As above	As above	As above	As above
(Brunner et al., 2013)/ Lilly Safety Database for olanzapine/ Global/ Sept 1986 –Dec 2010 [34]	102 infants exposed while BF. Data collected by manufacturer from spontaneous reports, clinical trials, and post-marketing studies.	High No information on covariates or confounding.	Unknown Minimised by searches of databases and the worldwide literature for reports of exposure and adverse events.	Low Only verified reports used.	Unknown Medical information sought, but concomitant exposures not reported.	High 102 under-estimates the extent of global olanzapine use.	Low Reports from the literature.	High Spontaneous and literature reports underestimate prevalence.	Moderate risk Important risks identified. Database funded by manufacturer.
(Gilad et al., 2011)/ Beilenson Information Service/ Israel/ Mothers seeking info 2005–2008 contacted 1–2 years after initial query (olanzapine) [36]	88 women 37 of 70 exposed to olanzapine (22 BF, 15 not BF) were followed up. 51 matched controls exposed to paracetamol.	Moderate Paracetamol control group were matched to the BF olanzapine group. Confounding by indication.	High Self-selected sample of women contacting an information centre.	Unknown Self-report of medicine use.	Unknown Self-report of concomitant medicine use.	Unknown Self-selected sample.	Unknown. Women self-reported.	Moderate Women’s self-reports may have overlooked subtle signs or exaggerated problems.	Moderate risk A relatively high number of problems in the 22 infants but confounding by indication cannot be discounted.
Goldstein et al., 2000)/ Manufacturer’s Worldwide Pharmacovigilance Safety Database for olanzapine/ all identified maternally exposed cases with outcomes reported until October 1998. [31]	2 breastfeeding exposures were identified retrospectively. Data collected by Eli Lilly from spontaneous reports, clinical trial cases, and post-marketing observational studies.	High Sample too small	High	Low risk for the 2 subjects.	Low Concomitant prescriptions reported.	High Only 2 breastfeeding cases were reported worldwide. This is an under-estimate.	Low Data from literature	High Reporting incomplete.	High risk Too few cases identified to give a useful overview. Funded by manufacturer.
	Single group of medicines								
Noseda et al 2021 Vigibase, Uppsala monitoring centre, Sweden / WHO database of spontaneous reports/ inception to end 2019 [32]	94 safety reports for monoclonal antibodies medicines in question, 1 of poor breastfeeding.	Unknown Too few data to assess	Low risk within the database. High risk from self-selected reporting of adverse events by women and professionals.	Low Medicines exposure reports accurately checked.	Low Reports were checked for other migraine medications.	High It is unlikely that only 1 case of ‘poor breastfeeding’ arose in worldwide exposure.	Unknown Many databases report to Vigibase, each making their own assessments of breastfeeding.	High ~5% ADRs are spontaneously reported. Many women and professionals do not spontaneously report ‘poor breastfeeding’.	High risk of missing data on breastfeeding.
Vieby et al 2013 MoBa /Norway/ mid-1999 to Dec 2008 (AEDs) [28]	223 women using AEDs from MoBa cohort, linked with national medicines databases. 276 women with epilepsy and no AED. 77,770 referents.	Low Considered in analyses	High Volunteer sample Volunteer, selection and collider bias.	Low Routine data used	Low Routine healthcare and prescription data used.	Moderate Good follow up, but cohort recruited only a sample of the population.	Low Standardised assessments	Low Comprehensive data collection	Moderate risk Important, if small, study.
(Gorman et al., 2012) / Information Service Clinical Research Program (CTIS) / California, US/ January 1st 2000 to June 1st, 2010 [37]	466: 167 exposed to SSRIs at birth, 117 exposed earlier in pregnancy, and 182 not exposed and enquiring about paracetamol or dental treatment.	Moderate Demographics accounted in analyses. Confounding by indication not accounted.	High Self-selected sample of women contacting an information centre and completing telephone follow up.	Unknown Women self-reported.	Unknown Women self-reported.	Unknown Self-selection and self-report.	Unknown. Women self-reported.	Moderate Self-report may have over-looked problems.	Moderate risk. An important study from a volunteer population.

Domains as in Sterne et al. 2016. We have been unable to assess bias due to deviation from intended intervention, as we did not locate any intervention studies.

## Discussion

The data available from databases and cohorts are too sparse to justify any firm conclusions, beyond the need for more data. Similarly, a scoping review of post-marketing studies identified only 10 studies reporting infant exposure during breastfeeding [65]. The absence of data from whole-population databases compounds concerns:

- Serious inter-generational ADRs from exposure via breastmilk are unquantifiable, but appear to be rare; however, concerns remain, particularly for medicines acting on the central nervous system (CNS).
- There is sufficient information to warrant frequent detailed monitoring of infants exposed via breastmilk, above and beyond routine ‘well-baby’ checks. There are no data to indicate that infant monitoring is unnecessary.
- For some medicines, there is insufficient information to advise patients whether the benefits of breastfeeding outweigh the harms from exposure via breastmilk.
- The more insidious but more pervasive harm of reduced breastfeeding rates following medicines exposure in late pregnancy, labour and *peri-partum* will remain unquantifiable until whole-population database and hospital prescribing pharmaco-epidemiological studies are undertaken.

The ADRs and harms to infants identified here reflect those reported in comprehensive reviews [66] of case series, small cohorts, databases [4, 67], and manufacturers’ literature. Two of 10 databases identified focused on olanzapine: two papers from the same database were sponsored by the manufacturers. The later paper, [34] is more reassuring than the earlier paper [31], but indicates that exposure during breastfeeding adversely affects 15.6% (16/102) infants, without reporting recovery or long-term outcomes. Concerns are supported and extended to risperidone and quetiapine in a small cohort [48]. This underlies the firm advice of manufacturers and the British National Formulary [68]. We identified very little data for alternative second-generation antipsychotics, and none for aripiprazole, where the BNF states ‘manufacturer advises avoid’, rather than simply ‘avoid–present in milk’ (no.83 p.430).

The benefits of breastfeeding to the infant, despite exposure to medicines for epilepsy *via* breastmilk, were apparent in the cohort with detailed long-term follow up [28], and other observational work; however, more data are needed for some AEDs, particularly phenytoin [69], ethosuximide, phenobarbital or primidone [70]. Further exploration is essential to review the benefit/harm balance, as other databases report only ‘gross motor development’ [35], rather than the full range of outcomes.

We have little information as to why breastfeeding rates were lower amongst those using prescription medicines or why people discontinued breastfeeding. However, the lower rates are consistent with those reported elsewhere [71, 72], and may be influenced by the absence of large studies offering reassurance of safety [73, 74] or the serious adverse effects reported in a small number of infants [30].

### Wider implications: The information desert

This review, like others [73], identified that exposures to medicines were associated with reduced breastfeeding rates. It also indicated that other exposures, such as recreational drugs [29, 39], may have a similar effect, suggesting that these exposures should be accounted in observational studies.

Currently, manufacturers are not obliged to provide data on breastfeeding. Data from animal studies are of uncertain value, as milk composition, and hence drug transfer, differ between species [60, 75]. The U.S. Food and Drug Administration (FDA) asks manufacturers to provide data on the impact of medicines on breastfeeding, and the breastfed infant, but this is not mandatory and there is an option for the ‘lactation’ section of product information to be omitted [76]. Current Medicines and Healthcare products Regulatory Agency (MHRA) guidance on UK product labelling in lactation indicates: ‘If available, clinical data from exposed breastfed infants should be mentioned as the conclusions of kinetic studies’ ([77] p.11). The European Medicines Agency (EMA) suggests that studies on breastfeeding ‘could be considered’, whilst noting, as indicated in this review, that ‘Reliable information regarding patient exposure in breastfeeding is not routinely available but may exist in some European birth cohorts.’ ([78] p.22). Alongside calls for further pharmacokinetic and pharmacogenomic studies [75], the concerns raised by the existing databases and cohorts should stimulate change in the availability of full-population databases with breastfeeding data [1, 79] (Yalcin et al 2022).

### Limitations of the data

Signals were generated by the studies in this review, but not pursued. Most authors based the associations reported on biological plausibility [80], rather than effect estimates: the *corpus* of literature supports the supposition that some infants may be vulnerable to the known ADRs of medicines transferred via breastmilk, but to an unknown and unpredictable extent. There was little information on dyads: most ADRs were reported in term infants or without specified gestation. The reduced renal function and impaired drug clearance in preterm infants [81, 82] suggests that omission of this vulnerable group may lead to under-reporting of harm.

No assessments of data quality were provided, and these are reported to be generally lacking even in large databases [83, 84]. Only one cohort [40] and six databases [28, 29, 34, 35, 37, 38] had >200 infants with breastfeeding data, the minimum sample size to detect serious adverse events in neonates [85]. It is estimated that spontaneous reports identify some 5% of ADRs [86], and the”less serious” more insidious reactions are particularly vulnerable to under-reporting [87]. This suggests that a more comprehensive approach to data collection is needed than provided in existing databases [30, 31, 34]. We have no indication as to the impact of any recall bias, volunteer selection bias, or social desirability response bias. These may over-estimate the prevalence of breastfeeding and under-estimate harms, which are over-represented in the most disadvantaged sections of the population [71, 88].

Like all non-randomised studies, those identified were vulnerable to unmeasured confounding, including unknown or lurking variables [89], and confounding by indication [90]. Selection, volunteer and collider bias impacts on studies that are not ‘whole-population’ [1, 12], including the databases identified here (Table 2). Their findings cannot be automatically transferred to the sections of the population who did not participate, mainly the economically disadvantaged [9], and recruitment by self-selection can distort associations *via* collider bias [12]. For example, when exploring the impact of medicines on initiation or duration of breastfeeding, if recruitment to the database or cohort were to favour participants who were both using medicines and breastfeeding, these characteristics would be over-represented. This over-representation would distort the sample and generate associations between breastfeeding and medicines exposure that may not appear in the wider (non-volunteer or unselected) population (1). Accordingly, the cohorts and most databases identified in this scoping review are not suitable for estimating the prevalence of infant ADRs arising from breastfeeding: rather, they alert professionals and families to potential problems to be monitored. Absence of hospital prescribing data may have caused exposure misclassification, and studies focusing on people contacting information services include only healthy survivors (immortal time bias) [91].

Infant follow-up ranged from 2 weeks to 3 years. No education outcomes were reported. Of the large studies, only the MoBa study systematically reported long-term neurodevelopmental outcomes. Twelve studies reported various and disparate developmental outcomes and assessments.

### Strengths and limitations of the review

This systematic review used a ‘wide-net’ approach to locate the primary surveillance data and identify the range of safety endpoints for a defined population, rather than focussing on a single safety endpoint [92] or information on each medicine category [67]. However, the terms “product surveillance” and “drug” identified articles on pesticides and recreational drugs. Study eligibility criteria, identification and selection of studies, data collection, appraisal, and synthesis were debated by all authors [92, 93], with due consideration for the differences between scoping reviews aiming to identify sources of data and systematic reviews aiming to answer clinical questions (S1 Checklist). The focus of the review on identifying databases with data on medicines AND infant outcomes AND breastfeeding led to omission of studies assessing only breastfeeding rates following medicines exposure [71, 72, 88], and some that did not use databases [37, 94]. Of the five European databases known to us as holding breastfeeding data, only the French databases appeared in our search [1].

Although included in the initial search strategy, papers in languages other than English (without English abstracts) were excluded, due to absence of tables of empirical data, and practical difficulties. We were unable to obtain some early papers, but none appeared to contain empirical data. We excluded studies on transfer of medicines through breastmilk where there was no information on infant outcomes.

In this scoping review, as anticipated, data on breastfeeding did not lend themselves to meta-analysis: outcomes, reporting methods, and study designs were heterogeneous. This complicated selection of a ‘risk of bias’ assessment instrument; however, since all studies related to exposure, we selected ROBINS-E, which is designed for studies of exposure [24, 95]. However, measurement of direction of bias was impractical [95]. Risk of bias was reported to illustrate the heterogeneity and paucity of the evidence rather than to influence the summary of the data [20]. Accordingly, our data summary, by failing to offer reassurance, serves to signpost the need for further research.

## Implications

### 1. Breastfeeding dyads

Professionals caring for breastfeeding patients receiving prescription medicines should monitor breastfed infants for signs of known ADRs: for example, those using mental health medicines should observe for sleepiness, drowsiness and sedation, and, where necessary, venous blood samples should be arranged [61, 96]. Monitoring ranges from checking the infant’s mouth for oral thrush to weight and sleep charting to venous blood sampling. Although risks are unquantified, due to lack of data, developmental and physical monitoring needs to be more intensive than the standard 72 hours and 6 week physical assessments [97]. Excessive sleeping will impair optimal development and may lead to failure to gain weight and thrive. These signs and symptoms are subtle, and may be overlooked if not specifically monitored. A quiet infant that cries little and sleeps a lot may be viewed as easy to manage, particularly where cultural norms suggest: ‘a sleeping baby is a good baby’.

Breastfeeding patients prescribed mental health medicines or antiepileptics are at increased risk of ‘not breastfeeding’ and early discontinuation. Professionals should be aware of this risk, and advise and support accordingly. Whilst failure to initiate breastfeeding may be related to choice (often driven by worry and uncertainty regarding transfer of medicines), early discontinuations is unlikely to be attributable to confounding by indication, and should be recognised as a possible biological effect of prescribed medicines on milk supply.

### 2. Paucity of data

This review has identified few ongoing databases with breastfeeding data, and none reporting prescribing in hospitals. Any infant harms due to exposure via breastmilk are likely to be subtle, rendering the absence of long-term follow-up and educational outcomes critical. Families and professionals rely on established databases, such as LactMed, for information [67]; however, despite thorough searches, the databases often have little information to offer, and report only small case series. No sections of the population should be excluded from the protection afforded by timely collection and analysis of data on the safety of medicinal products [78]. However, the omission of breastfeeding data from most population databases indicates that there are few data to inform breastfeeding patients and those intending to breastfeed a) whether lactation will be affected by prescription medicines, and b) how medicines will affect breastfed infants. To return investment in population healthcare databases pharmacoepidemiologists should have good quality data to explore any relationships between medicines exposures, breastfeeding and short- and long-term infant outcomes.

## Supporting information

S1 File Registry of systematic reviews. Review no.994.
(HTML)

S1 Table Cohort studies in chronological order. (DOCX)

S1 Checklist PRISMA-ScR checklist. (DOCX)
