# Supplementary material for: Where are the data linking infant outcomes, breastfeeding and medicine exposure? A systematic scoping review
Source: PLoS One. 2023 Apr 26;18(4):e0284128. doi: 10.1371/journal.pone.0284128 (PMC10132552; doi:10.1371/journal.pone.0284128)
Supplement: S1 File — Review no.994. (HTML) [file pone.0284128.s001.html]

   Browse the Registry - Research Registry      

This website uses cookies to ensure you get the best experience on our website. Learn more

Got it!

Research Registry

- Why Register
- About

  - About the Research Registry
  - The Team
  - Data Curation
  - Quality Indicators
  - Publications and Presentations
  - News
  - Testimonials
  - Editor’s Choice
  - Who is registering?
- Browse the Registry
- Register Now

  - Register Your Research Study
  - Register Your Systematic Review
- Help & Support

  - The Research Registry Guidebook
  - FAQS
  - Terms and Conditions

# Browse the Registry

- Research Registry
- Registry of Systematic Reviews/Meta-Analyses

Registry of Systematic Reviews/Meta-Analyses


Registry of Systematic Reviews/Meta-Analyses Details

Print

## Registry of Systematic Reviews/Meta-Analyses Details

Registration Date

September 11, 2020 14:00

Review Registry Unique Identifying Number

reviewregistry994

Title

Review of data sources for breastfeeding and medicine exposure.

Key Review Question and Objectives

The current review aims to create a summary of data sources with information  
on breastfeeding and medicines exposure either before or during lactation.

Lay Summary (please do not just paste your abstract. Please summarise your research, written in a way the public can understand)

This review aims to find data sources such as databases with information on  
individuals who were breastfeeding and also had exposure to medicines.

Does this Systematic Review include a Meta-Analysis?

No

Primary Investigator

Sue Jordan

Other Investigator(s)

Sophia Komninou

Additional Investigators

Naomi Marfell

Participating Institution(s)

Swansea University  
Swansea  
SA2 8PP

Contact Details for Public/Scientific Enquiries (include postal address)

College of Human and Health Sciences, Swansea University, SA2 8PP

Primary Email

n.r.marfell@swansea.ac.uk

Secondary email

s.e.jordan@swansea.ac.uk

Telephone number for enquiries

01792 205678

Countries involved

Worldwide

Funding

The publication is part of the activities within the ConcePTION project. It has  
received funding from the Innovative Medicines Initiative 2 Joint Undertaking  
under grant agreement No 821520. This Joint Undertaking receives support from  
the European Union's Horizon 2020 research and innovation programme and  
EFPIA.

Search Strategy

The following electronic databases and data sources will be searched using a combination of controlled vocabulary (MeSH) and free text terms.   
Database Sources Searched:  
PubMed/Medline   
Scopus  
CINAHL   
PsycINFO  
Web of Science  
British Nursing Database  
Proquest  
Drugs and Lactation Database (LactMed)   
ZETOC  
TRIP  
MIDIRS  
Wiley Online Library   
  
Data Sources:  
EUROmediSafe Inventory  
Other data sources  
Search terms:  
“Breastfeeding OR Lactation OR Breastfe\* OR Breast-fe\* OR “Breast fe\*” OR Lactat\* OR “Infant feed\*” OR “Infant Nutrition”   
AND  
“Pharmacovigilance OR Product Surveillance OR Postmarketing OR Drug Monitoring OR Adverse Drug Reactions OR Pharmacovigilan\* OR “Drug monitor\*” OR “Postmarketing Surveillance” OR “Post-marketing Surveillance” OR “Post marketing Surveillance” OR “Adverse Drug Reaction\*”  
NOT  
For two databases (PubMed and PsychINFO) it was necessary to specify “NOT economics” in order to obtain more relevant results.  
The search:  
• No language restrictions  
• Data search restricted to papers reporting on humans only  
• EU and Worldwide search   
• Search will go as far back as possible, to, if possible, capture the emergence of the topic  
• A hand search (quick screening of titles and abstracts) is to be done during the search (before adding sources to the libraries) with a further screening to be done by another researcher

Information Sources (describe all information sources (e.g., databases with dates of coverage, contact with study authors to identify additional studies) in the search and dates you will or last searched)

The following electronic databases and data sources will be searched using a combination of controlled vocabulary (MeSH) and free text terms.   
Database Sources Searched:  
PubMed/Medline   
Scopus  
CINAHL   
PsycINFO  
Web of Science  
British Nursing Database  
Proquest  
Drugs and Lactation Database (LactMed)   
ZETOC  
TRIP  
MIDIRS  
Wiley Online Library   
  
Data Sources:  
EUROmediSafe Inventory  
Other data sources  
Search terms:  
“Breastfeeding OR Lactation OR Breastfe\* OR Breast-fe\* OR “Breast fe\*” OR Lactat\* OR “Infant feed\*” OR “Infant Nutrition”   
AND  
“Pharmacovigilance OR Product Surveillance OR Postmarketing OR Drug Monitoring OR Adverse Drug Reactions OR Pharmacovigilan\* OR “Drug monitor\*” OR “Postmarketing Surveillance” OR “Post-marketing Surveillance” OR “Post marketing Surveillance” OR “Adverse Drug Reaction\*”  
NOT  
For two databases (PubMed and PsychINFO) it was necessary to specify “NOT economics” in order to obtain more relevant results.  
The search:  
• No language restrictions  
• Data search restricted to papers reporting on humans only  
• EU and Worldwide search   
• Search will go as far back as possible, to, if possible, capture the emergence of the topic  
• A hand search (quick screening of titles and abstracts) is to be done during the search (before adding sources to the libraries) with a further screening to be done by another researcher

Inclusion Criteria

• Empirical research articles and grey literature reporting primary research, published in full, including all research methods (but excluding reviews)  
• Includes human sample  
• Includes breastfeeding or ‘infant feeding’ as an outcome, predictor or covariate  
• Includes some details of medicine exposure either before or during lactation

Exclusion Criteria

Animal studies  
• No data on infant feeding / breastfeeding  
• No data on medicines (if any) prescribed, administered or ingested

Condition, disease or problem being studied

Human lactation  
Studies including breastfeeding and medicine exposure

Patients/Participants/Population

Human participants who are breastfeeding or are recorded as not  
breastfeeding and have records of exposure or non-exposure to medicine  
either before or during lactation.

Intervention(s) or Exposure(s)

Exposure to any medication before or during lactation

Control or Comparator(s)

The range of studies included will be kept broad to try and identify all sources of  
information on breastfeeding and exposure to medicines. Studies with and  
without a control group will be included. Lactation with and without  
documented exposure to medicines will be compared.

Primary Outcomes

We are aiming to create a summary of data sources relating to breastfeeding  
and exposure to medicines. Outcome of studies will include information about  
breastfeeding and medicines although it is not necessary that included studies  
tie the two together.

Secondary Outcome(s)

1. We shall characterise the data by:  
Outcomes reported  
• definitions of breastfeeding  
• infant age at recording of infant feeding  
exposures reported  
• names of medicines (coding system)  
• doses   
• timing of exposure: trimester, labour, lactation  
covariates reported  
• socio-economic status  
• co-exposures, including smoking, substances  
• indications  
• other, including gestation, growth, mode of delivery, maternal age, BMI  
proportion of missing data  
2. We shall describe any reports of the effect of prescribed medicines on breastfeeding. We anticipate that the data will be too heterogeneous for any meta-analyses.

Data extraction (selection and coding)

Following removal of duplicates, publications will be screened by titles and abstracts to identify those which meet the study inclusion criteria. Publications will be screened independently by 3 researchers (NM, SJ, SK) in accordance with the pre-specified inclusion and exclusion criteria.  
Articles which are not relevant to the study topic, and clearly fall outside the study focus, will be excluded. The results will be shared between the researchers. Any discrepancies will be resolved by discussion and the involvement of a third reviewer where necessary.   
   
Full texts of all included articles, will then be retrieved, read, and appraised, using a data extraction form.   
Details of all included reviews will be extracted into predefined tables with the following information extracted:   
• Sample characteristics (size, attrition, country of study),   
• Data source  
• Summary of the data included

Risk of bias (quality) assessment

This study aims to identify the sources of data and as such studies will not be  
assessed for risk of bias. Proportion of missing data will be reported. Risk of  
selection bias will be commented, if possible

Data Synthesis Strategy

This study aims to identify the sources of data, a meta-analysis of the data is not planned. A narrative summary of sources of data will be presented  
Following extraction, a preliminary synthesis will undertaken in tabular form, studies will be searched and a framework constructed with the following details:  
• Study objectives  
• Country of study  
• Details of the data source  
  
The study characteristics will be described narratively.  
If outcome data are available, these will be reported including.   
• Sample characteristics (country of study, sample size, proportion of population),   
• Timeframe of study  
• Details relating to breastfeeding (exclusive, partial)  
• Age of the infant  
• Any demographics of the diad as available  
• Drugs (name/ATC code/ dates of drug exposure/ long term vs short exposure)  
• Data source

Analysis of subgroups or subsets

None proposed

Dissemination plan

The publication is part of the activities within the ConcePTION project. The  
research will be disseminated as part of this project and in relevant  
publications.

Stage of Review at Time of Submission

Protocol Written, Preliminary searches, Piloting of the study selection process

Post Review Results (once available)

User

Back to Registry of Systematic Reviews/Meta-Analyses

- Research Registry
- Registry of Systematic Reviews/Meta-Analyses

Register Your Study Now Browse the Registry

- © 2022 Research Registry
- Privacy Policy
- Disclaimer
- Contact
- Connect with us:
