# Supplementary material for: Where are the data linking infant outcomes, breastfeeding and medicine exposure? A systematic scoping review
Source: PLoS One. 2023 Apr 26;18(4):e0284128. doi: 10.1371/journal.pone.0284128 (PMC10132552; doi:10.1371/journal.pone.0284128)
Supplement: S1 Table — (DOCX) [file pone.0284128.s002.docx]

**S2 Supplementary Table A : Cohort studies in chronological order.**

| **Reference/ Location** | **Numbers in study** | **Study Objectives** | **Meds used** | **Doses** | **Impact on breast-feeding** | **Impact of medicine on breastfeeding infant** | | **Timing of medicine use** | |  |  |
| --- | --- | --- | --- | --- | --- | --- | --- | --- | --- | --- | --- |
| 1. Gehrmann et al 2021(1) / Germany, specialist outpatient clinics [61] | 3 | To evaluate the development of infants exposed to lithium salts via breastmilk and the need for infant monitoring | Lithium carbonate plus co-prescriptions for hypothyroidism (3) and depression (2) | Lithium doses 225-1400mg. Doses were titrated for all participants during pregnancy and the puerperium. Levothyroxine doses stated for 1 participant (100mcg). | Not reported | Development, thyroid and renal function reported as normal. | | Pre-conception, pregnancy, puerperium, breastfeeding | |  |  |
| 1. Sinha et al 2021 (2)/ India, mother and baby in-patient mental health unit + 1-3 month follow up. [48] | 28 (4 fully BF, 24 partially BF), 17 returned for follow-up | To report any adverse effects of exposure to atypical antipsychotics to full-term, breastfed infants. | Olanzapine (15), risperidone (11), quetiapine (2). Co-exposures included chlorpromazine, lorazepam, phenytoin, trihexypjrnidyl. | Olanzapine 2.5-30mg/day  Risperidone 4-8mg/ day  Quetiapine 25-200 mg / day. | Not reported | | Olanzapine or risperidone:  Sedation (3/28)  Constipation (2/28)  Diarrhoea (2/28)  Developmental delay in 5/17 infants (all 3 antipsychotics), 4 not exposed to medicines in pregnancy. A further 4 infants were <3^rd^ centile for weight or height (one exposed to phenytoin prenatally), and 1 infant was both small and delayed. | | Acute episodes whilst breastfeeding.  2/17 followed up were exposed to quetiapine or phenytoin *in utero. in utero.* | |  |
| 1. Moroni et al., 2019 (3)/ Buenos Aires   Children’s Hospital, Buenos Aires, Argentina [55] | 10 infants aged 1-11 months | To prospectively study nifurtimox (antiprotozoal for Chagas disease) transfer into breast milk in a cohort of lactating patients with Chagas disease to explore safety.  To develop evidence-based recommendations for management of CD during lactation. | Nifurtimox | Breastfeeding patientswith Chagas disease received 8.3 to 12 mg/kg/day (120 mg tablets) (Lampit, Bayer, El Salvador), for 30 days. | Not reported : 3 infants were exclusively BF. 6/10 women reported ADRs, including eosinophilia, emesis + fever, headache, and psychomotor agitation. No ADRs were considered serious. No infant stopped breastfeeding. | | All infants were healthy during and after the study, as assessed by paediatricians skilled in the evaluation of paediatric patients with CD. No ADRs were observed in the breastfed infants. There were no changes in their behaviour, weight, progress or other effects potentially attributable to BF. | | Not taken during pregnancy  Used during lactation | | |
| 1. Lam et al., 2013(4)/Toronto, Canada [47] | 67 infants exposed to oxycodone | To assess the effect of maternal CYP2D6 CYP3A5, ABCB1 and OPRM1 polymorphisms in predicting both neonatal and maternal CNS depression after oxycodone use during lactation. | Oxycodone | Mean doses 0.24mg/kg/day [SD 0.19] for mothers of infants with CNS depression and 0.20 mg/kg/day [SD 0.14] for mothers of infants without CNS depression. | Not reported | | Symptomatic infants displayed sleepiness or lethargy during drug exposure and reversible CNS depression on discontinuation of oxycodone or BF, as reported by mother.  Mothers of symptomatic infants used oxycodone for more BF days than mothers of asymptomatic infants. | | Not used during pregnancy.  Used during lactation | | |
| 1. Kelly et al., 2012(5)/ Hospital for sick children in Toronto, Ontario [44] | 124 infants exposed to benzodiazepines during BF. | To assess central nervous system (CNS) depression and other adverse effects in infants exposed to benzodiazepines through breast milk. | Benzodiazepines including lorazepam, clonazepam, and midazolam.  Other CNS depressants were co-prescribed (more so where infants were sedated).. | Reported for the 2 infants with CNS depression:  1) 0.25 mg of alprazolam on 2 occasions + 50 mg of sertraline daily + 2.5 mg of zopiclone when necessary.  2) long-term use: 0.25 mg clonazepam bd + 1 mg of flurazepam daily + 1 mg bupropion daily + 0.75 mg risperidone daily. | 32/124 mothers reported ADRs, mainly sedation, confusion or headache. Both sedated infants received formula supplementation, as did 58/122 unaffected infants. | | CNS depression. 2 cases amongst 124 infants. | | The sedated infant of the mother using benzodiazepine long-term was also exposed in utero. Both infants were exposed during lactation. (No details re which trimester) | | |
| 1. Dryden – Peterson et al., 2011 (6)/ Botswana /March 2001 -October 2003   Cohort formed by following up 2 RCTs. [40] | 1719  Mashi and Mma Bana MTCT prevention trials:  691 HAART (highly active anti-retroviral therapy)-BF, 503 ZDV (zidovudine)-BF, and 525 ZDV-FF (formula fed). | To evaluate the effect of maternal highly-active antiretroviral therapy (HAART)started in pregnancy on the incidence of anemia among HIV-exposed, uninfected infants participating in two clinical trials in Botswana. | HAART | Not reported | Not reported | | 118 infants had severe anaemia: 11 required transfusion.  By 6 months, 12.5% of HAART-BF infants experienced severe anaemia, compared with 5.3% of ZDV-BF and 2.5% of ZDV-FF infants. In adjusted analysis, HAART-BF infants were at greater risk of severe anaemia than ZDV-BF or ZDV-FF infants (adjusted odds ratios 2.6 and 5.8, respectively). | | All trimesters and during lactation. | | |
| 1. Chaves et al., 2009(7) / Maternity unit of Hospital Manoel Gonçalves de Sousa Moreira, city of Itaúna, state of Minas Gerais, Brazil [41] | 129 | To describe the frequency and type of self-medication by mothers, and the association between self-medication and duration of breastfeeding. | The medicines most frequently used were: analgesics/antipyretics (54.4%), non-steroidal anti-inflammatories (15%), spasmolytics (6.2%), laxatives (3.5%), benzodiazepines (3%), nasal decongestants (1.4%), antibiotics (0.9%), (others (15.6%)). The drugs most often used were dipyrone (31.5%) and paracetamol (17.9%). | Not reported | 6 women stopped BF, due to use of prescription medicines. Self-medication was not associated with BF cessation (RR = 1.59; 95%CI 0.81-3.11). | | No mothers reported adverse effects in infants | | Used during lactation  no data on whether the medication was also taken during pregnancy | | |
| 1. Newport et al., 2009 (8)/ Emory Women's mental Health Program, Georgia, US | 13 | To characterise the concentrations of venlafaxine and desvenlafaxine in breast milk and in nursing infants’ plasma, and their determinants. | Venlafaxine  Three participants were also receiving psychotropics at the time of sampling. | 2 nursing mothers were treated with immediate release venlafaxine . 11 received extended-release venlafaxine. Mean dose 194.3 mg/ day [95% CI 137.1-251.6mg/day], range 37.5mg/day to 300 mg/day | Not reported | | All infants reported healthy at delivery,  5 minute Apgar scores ≥f 8. 1 infant was admitted to a neonatal intensive care unit for18 hours due to tachypnoea, and was administered oxygen.  Three infants were born pre-term (36.1, 36.3, 36.3 weeks).  No adverse events were reported among either the mothers or their nursing infants. | | One mother initiated venlafaxine immediately after delivery, 12 nursing mothers received venlafaxine during pregnancy and post partum.  No information regarding trimesters. | | |
| 1. Viguera et al., 2007 (9)/ Perinatal and Reproductive Clinical Research Programme at Massachusetts General Hospital in Boston, US [56] | 10 | To quantify lithium exposure in nursing infants | Lithium | Mean maternal lithium dose 850 mg/day (SD = 220, range = 600-1200 mg/day). | Not reported | | No observable growth or developmental delays reported.  One infant had elevated TSH at 8 weeks; BF cessation was recommended. The mother decided to discontinue lithium and nurse her infant. 8 weeks later, TSH was normal.  Two other infants had elevated blood urea nitrogen without clinical signs of hypovolemia.  In a fourth infant, creatine rose over several months from 0.3 to 0.6 mg/dl and normalised a year later. | | Used in 3^rd^ trimester (in infant with elevated TSH).  Used during lactation. | | |
| 1. Moretti et al., 2006 (10)/ Canada | 4 | To report the clinical outcome of infants whose mothers were taking azathioprine while nursing and to quantify the transfer of 6-mercaptopurine (6-MP), its active metabolite, into breast milk. | Azathioprine. 2 infants co-exposed to warfarin *in utero.* | 100 mg/day, | Not reported | | Not reported  All 4 infants were healthy at birth.  2 of the infants were born at 36 weeks, and 1 was small for gestational age Upon follow-up no ADRs detected among the 4 infants. In the 2 cases in this series in which breastmilk was analyzed for 6-MP, concentrations of this potentially toxic metabolite were below the limit of detection. | | Used in all 3 trimesters of pregnancy, and  during lactation | | |
| 1. Johannessen et al., 2005 (11)/ Norway | 8 | To study the pharmacokinetics of levetiracetam (LEV) at birth, during lactation, and in the nursed infant. | Levetiracetam | Twice daily (dose not reported) | Not reported | | The infants exposed to LEV had a mean birth weight of 3,650g (range, 2,970–4,220 g) and appeared healthy throughout the study. | | Use not reported in pregnancy.  Used during lactation | | |
| 1. Rampono et al., 2006 (12)   Subiaco, Australia | 8 infants aged 2-4 months | To investigate the milk transfer, infant dose and safety of escitalopram during BF | Escitalopram | Median daily dose 10mg (range 10-20mg), taken for a median of 55 days before the study. | Not reported, lactation established. | | All infants exhibited normal behaviour and development milestones for age. | | No information on exposure during pregnancy  Used during lactation. | | |
| 1. Lee et al,. 2004 (13)/ Hospital for sick children, Toronto, Canada [43] | 31 exposed to citalopram + 31 matched comparators with no medical conditions + 5 women with unmedicated depression + 7 women prescribed another antidepressant. | To determine the frequency of adverse events in infants nursed by women receiving citalopram. | Citalopram | Mean (SD) 25.3 ±11.4 mg per day (range, 10-60 mg)  The mean duration of infant exposure to citalopram through breast milk at time of study was 4.8-3.9 months. | In one case of adverse events, BF was discontinued 2 weeks after the start of citalopram therapy.  Twenty of the 31 women were still breast-feeding while taking citalopram at follow-up. | | 3 of 31 infants exposed to citalopram experienced ADRs during BF (one case each of colic, decreased feeding, and irritability/ restlessness). For the infants with colic and decreased feeding, physician attention was sought, but there was no intervention.  For the infant with irritability and restlessness, symptoms started after the mother commenced citalopram at 2 months *post-partum*. BF was discontinued 2 weeks later, and the infant’s symptoms subsided. One infant exposed to another antidepressant had gastro-intestinal symptoms. | | No information on whether citalopram was also taken during pregnancy.  Used during lactation. | | |
| 1. Merlob et al., 2004 (14)/ Department of Neonatology, Rabin Medical Centre, Israel [49] | 27 breastfeeding and using paroxetine for 2 weeks + 19 not BF and not taking paroxetine + 27 BF mothers not taking any medicines. | To examine weight gain in infants BF by mothers taking paroxetine.  To assess the clinical implementation of recommendations of the teratology service in our centre. | Paroxetine | Average daily dose 20.7mg/day ±6.7 mg/day | Not reported | | No differences in weight gain at 6 and 12 months. Weight was slightly higher in BF infants not exposed to medication. One infant became irritable. No other adverse effects of maternal use of paroxetine were observed, by either maternal report nor medical follow-up. All infants reached the usual developmental milestones at 3, 6 and 12 months. | | Use in pregnancy varied.  Used during lactation. | | |
| 1. Berle et al., 2004 (15)/Bergen, Norway | 25 | To quantify the drug exposure in BF infants of antidepressant-treated mothers,  To identify possible adverse events,  To relate these variables to maternal and infant drug metabolism-relevant genotypes and milk triglyceride content. | citalopram (N = 9),  sertraline (N = 6),  paroxetine (N = 6),  fluoxetine (N = 1),  venlafaxine (N = 3) | Not reported | Not reported | | There was no evidence of ADRs s in the drug-exposed infants. | | Used during lactation | | |
| 1. Gardiner et al., 2003 (16) / King Edward Memorial and Princess Margaret Hospitals, Western Australia [27] | 7 | To characterize infant drug doses and breast-milk-to-plasma area-under-the-curve ratios for olanzapine.  To determine plasma concentrations and effects of this drug on breast-feeding infants at 2.4 months | Olanzapine | The median dose of olanzapine ingested by the women was 7.5 mg/day (range=5– 20) or 127 µg/kg/day (range=75–286). | Not reported | | Examinations of infants B, C, E, and F indicated no ADRs Infant E had no developmental delay.  Infant D had a developmental age of 78% of chronological age (Griffiths developmental assessment); causality was difficult to assess due to recent co-medication with clonazepam, sertraline, valproate, thioridazine, and droperidol.  Infant A was not assessed.  Infant G was referred for drowsiness, but was unaffected when assessed: the maternal olanzapine dose had been halved to 5 mg/day 3 weeks earlier.  Infants’ weights crossed the centiles. Infant D fell from 97^th^ centile at birth to 50^th^. | | All trimesters and during BF | | |
| 1. Heikkinen et al., 2002 (17) / Turku University Central Hospital, Finland [50] | 11 exposed to citalopram + 10 matched comparators | To monitor maternal, infant and breast milk concentrations of citalopram and its two main demethylated metabolites during pregnancy, delivery and lactation.  To characterize the effects of citalopram and its metabolites on developing foetuses and infants | Citalopram | 20 to 40 mg once daily | Not reported. Mothers in both groups (comparator and intervention) BF their infants. | | Development of all 21 infants was followed up to 1 year with no difference between the study groups in weight at 12 months (all within the normal range).  The neurological development of all infants was normal. One infant exposed to citalopram was not walking at 1 year, but walked at 18 months. | | Used in all trimesters of pregnancy and lactation. | | |
| 1. Yoshida et al., 1998 (18)/ Bethlem and Maudsley NHS Trust, London, UK [51] | 4 | to assess cognitive and psychomotor development of infants whose mothers took fluoxetine | fluoxetine | 20-40 mg/day | Reported for one infant: sucking was strong and muscle tone was normal. | | All infants were observed to be developing normally and showed no abnormal findings on neurological examination. | | Not used during pregnancy  Used during lactation. | | |
| 1. Yosida et al., 1997 (19)/ Bethlem Royal Hopsital, London, UK [54] | 10 BF dyads exposed to tricyclic antidepressants +15 matched dyads not BF | To compare BF and bottle-fed infants of mothers exposed to tricyclic antidepressants. | Antidepressants: imipramine, amitriptyline clomipramine and dothiepin. | Range from 50 mg/day to 225 mg/day | Not reported | | All infants, except BF infant 5, showed normal development, assessed using the Amiel–Tison neurological test.  Infant 5 scored as borderline, before the mother started medication. He was hypotonic before and throughout the medicated-breast-feeding period, but had no sucking problems and weight gain was normal.  All infants, except subject 5, were normal on the Amiel–Tison test. | | No data on pregnancy use  Used during lactation. | | |
| 1. Ito et al., 1995 (20)/ Ontario, Canada [52] | 68: 34 women using AEDs and 34 age-matched controls not using AEDs or any potentially teratogenic medicines, recruited from the Mother-risk programme and interviewed. | To characterize breast-feeding initiation and the duration of breast-feeding in women receiving antiepileptics. | Anti-epileptics (AEDs): carbamazepine, valproic acid, phenytoin, phenobarbital, ethosuximide, clobazam monotherapy or combinations. | Not reported | Fifty percent (17/34) of the group receiving antiepileptics initiated BF significantly less than controls (29/34). The 17 women using AEDs who chose BF terminated BF significantly earlier than did the control group (4.7 +/- 2.6 vs 9.3 +/- 5.7 months *post-partum*). | | No mothers reported any adverse effects to infants | | Pregnancy and lactation. | | |
| 1. Passmore et al, 1988 (21) / Belfast, UK [53] | 35 exposed to metronidazole  24 exposed to ampicillin alone  31 with no medication exposure | To monitor metronidazole and hydroxymetronidazole (metabolite) concentrations in maternal milk and plasma over several dosing periods (under maternal steady state conditions).  To determine infant plasma concentrations and to monitor infants for adverse reactions to maternal metronidazole therapy. | Metronidazole | Metronidazole  400mg three times daily | Not reported | | Oral candidiasis was reported in one infant (treated with nystatin and maternal drug therapy was suspended). The mother had no monilial infection.  The same fungal species was isolated from buccal and perianal swabs from an infant in the ampicillin control group with napkin rash. *Candida species* were isolated more often from infants in the metronidazole group than the non-drug group, and the growth was consistently heavier. Differences did not reach statistical significance. | | Not taken during pregnancy. Used during lactation. | | |
| 1. Boutroy et al., 1986 (22)/ Paris, France [46] | 7 | To evaluate the possible risk of exposure to beta-blockers in neonates BF by mothers treated with acebutolol. | Acebutolol | 0.2 to 1.2 g/day | Not reported | | Hypotension, bradycardia and  transient tachypnoea were observed in one infant. | | Use during pregnancy not detailed. Used during lactation. | | |
| 1. Froescher et al., 1984 (23) / Bonn, Germany [42] | 13 | To report on the concentrations of carbamazepine (CBZ) in breast milk and the infant's blood. | Carbamazepine (CBZ). Co-medications: 3 mothers received valproric acid, 2 primidone and one clonazepam. | No information | Weakness in suckling was observed in 1 of 15 infants whose mothers were using CBZ monotherapy. | | Formula-fed infants of mothers receiving combination therapy gained weight more rapidly than mixed fed infants. This was attributed to poor suckling and vomiting. | | Use in pregnancy not reported.  Used during lactation. | | |
| 1. Chinnatamby, 1973 (24) / Ceylon [45] | 134 (original cohort)  21 randomly selected for the study | To report the effects of a combination of 50mcg ethinyloestiradiol plus 250 mcg. D-norgestrel on lactation. | Combination of ethinyloestiradiol plus. D-norgestrel | 50mcg ethinyloestiradiol 250 mcg. D-norgestrel | Of the 21 women selected at random, 18 reported no change in milk production and continued to lactate until the end of treatment.  Lactation decreased in the other 3 women. | | No mothers reported adverse effects in infants | | Not taken during pregnancy  Used during lactation | | |

Notes to table: BF – breastfeeding, RR – relative risk

Some, not all, studies are referred to in the manuscript, and their reference numbers are included here. All studies are referenced in the list below.

## References for this table

1. Gehrmann A, Fiedler K, Leutritz AL, Koreny C, Kittel-Schneider S. Lithium medication in pregnancy and breastfeeding—a case series. Medicina. 2021;57(6):634.

2. Sinha SK, Kishore MT, Thippeswamy H, Kommu JVS, Chandra PS. Adverse effects and short-term developmental outcomes of infants exposed to atypical antipsychotics during breastfeeding. Indian Journal of Psychiatry. 2021;63(1):52.

3. Moroni S, Marson ME, Moscatelli G, Mastrantonio G, Bisio M, Gonzalez N, et al. Negligible exposure to nifurtimox through breast milk during maternal treatment for Chagas Disease. PLoS Neglected Tropical Diseases: Public Library of Science; 2019. p. e0007647.

4. Lam J, Kelly L, Matok I, Ross CJD, Carleton BC, Hayden MR, et al. Putative association of ABCB1 2677G>T/A with oxycodone-induced central nervous system depression in breastfeeding mothers. Therapeutic Drug Monitoring2013. p. 466-72.

5. Kelly LE, Poon S, Madadi P, Koren G. Neonatal benzodiazepines exposure during breastfeeding. Journal of Pediatrics: Mosby; 2012. p. 448-51.

6. Dryden-Peterson S, Shapiro RL, Hughes MD, Powis K, Ogwu A, Moffat C, et al. Increased risk of severe infant anemia after exposure to maternal HAART, Botswana. Journal of Acquired Immune Deficiency Syndromes2011. p. 428-36.

7. Chaves RG, Lamounier JA, César CC. Automedicação em nutrizes e sua influência sobre a duração do aleitamento materno. Jornal de Pediatria: Sociedade Brasileira de Pediatria; 2009. p. 129-34.

8. Newport DJ, Ritchie JC, Knight BT, Glover BA, Zach EB, Stowe ZN. Venlafaxine in human breast milk and nursing infant plasma: Determination of exposure. Journal of Clinical Psychiatry: Physicians Postgraduate Press, Inc.; 2009. p. 1304-10.

9. Viguera AC, Newport DJ, Ritchie J, Stowe Z, Whitfield T, Mogielnicki J, et al. Lithium in breast milk and nursing infants: Clinical implications. American Journal of Psychiatry: American Psychiatric Association; 2007. p. 342-5.

10. Moretti ME, Verjee Z, Ito S, Koren G. Breast-feeding during maternal use of azathioprine. Annals of Pharmacotherapy: SAGE PublicationsSage CA: Los Angeles, CA; 2006. p. 2269-72.

11. Johannessen SI, Helde G, Brodtkorb E. Levetiracetam concentrations in serum and in breast milk at birth and during lactation. Epilepsia: John Wiley & Sons, Ltd; 2005. p. 775-7.

12. Rampono J, Hackett LP, Kristensen JH, Kohan R, Page‐Sharp M, Ilett KF. Transfer of escitalopram and its metabolite demethylescitalopram into breastmilk. British journal of clinical pharmacology. 2006;62(3):316-22.

13. Lee A, Woo J, Ito S. Frequency of infant adverse events that are associated with citalopram use during breast-feeding. American Journal of Obstetrics and Gynecology: Mosby; 2004. p. 218-21.

14. Merlob P, Stahl B, Sulkes J. Paroxetine during breast-feeding: Infant weight gain and maternal adherence to counsel. European Journal of Pediatrics: Springer; 2004. p. 135-9.

15. Berle JØ, Steen VM, Aamo TO, Breilid H, Zahlsen K, Spigset O. Breastfeeding during maternal antidepressant treatment with serotonin reuptake inhibitors: Infant exposure, clinical symptoms, and cytochrome P450 genotypes. Journal of Clinical Psychiatry: Physicians Postgraduate Press, Inc.; 2004. p. 1228-34.

16. Gardiner SJ, Kristensen JH, Begg EJ, Hackett LP, Wilson DA, Ilett KF, et al. Transfer of olanzapine into breast milk, calculation of infant drug dose, and effect on breast-fed infants. American Journal of Psychiatry: American Psychiatric Publishing; 2003. p. 1428-31.

17. Heikkinen T, Ekblad U, Kero P, Ekblad S, Laine K. Citalopram in pregnancy and lactation. Clinical Pharmacology and Therapeutics: John Wiley & Sons, Ltd; 2002. p. 184-91.

18. Yoshida K, Smith B, Craggs M, Channi Kumar R. Fluoxetine in breast-milk and developmental outcome of breast-fed infants. British Journal of Psychiatry: Cambridge University Press; 1998. p. 175-9.

19. Yoshida K, Smith B, Craggs M, Kumar RC. Investigation of pharmacokinetics and of possible adverse effects in infants exposed to tricyclic antidepressants in breast-milk. Journal of Affective Disorders: Elsevier; 1997. p. 225-37.

20. Ito S, Moretti M, Lian M, Koren G. Initiation and duration of breast-feeding in women receiving antiepileptics. American Journal of Obstetrics and Gynecology: Mosby; 1995. p. 881-6.

21. Passmore C, McElnay J, Rainey E, D'Arcy P. Metronidazole excretion in human milk and its effect on the suckling neonate. British Journal of Clinical Pharmacology: John Wiley & Sons, Ltd; 1988. p. 45-51.

22. Boutroy MJ, Bianchetti G, Dubruc C, Vert P, Morselli PL. To nurse when receiving acebutolol: Is it dangerous for the neonate? European Journal of Clinical Pharmacology: Springer; 1986. p. 737-9.

23. Froescher W, Eichelbaum M, Niesen M, Dietrich K, Rausch P. Carbamazepine levels in breast milk. Therapeutic Drug Monitoring: Ther Drug Monit; 1984. p. 266-71.

24. Chinnatamby S. Effects of 'nordiol' on fertility and lactation: Some preliminary observations. Current Medical Research and Opinion: Taylor & Francis; 1973. p. 376-8.
